# The PRINTO evidence-based proposal for glucocorticoids tapering/discontinuation in new onset juvenile dermatomyositis patients

**DOI:** 10.1186/s12969-019-0326-5

**Published:** 2019-05-22

**Authors:** Gabriella Giancane, Claudio Lavarello, Angela Pistorio, Sheila K. Oliveira, Francesco Zulian, Ruben Cuttica, Michel Fischbach, Bo Magnusson, Serena Pastore, Roberto Marini, Silvana Martino, Anne Pagnier, Christine Soler, Valda Staņēvicha, Rebecca Ten Cate, Yosef Uziel, Jelena Vojinovic, Elena Fueri, Angelo Ravelli, Alberto Martini, Nicolino Ruperto, for the Paediatric Rheumatology International Trials Organisation (PRINTO)

**Affiliations:** 1 IRCCS Istituto Giannina Gaslini, Clinica Pediatrica – Reumatologia, PRINTO, Genoa, Italy; 2 IRCCS Istituto Giannina Gaslini, Servizio di Epidemiologia e Biostatistica, Genoa, Italy; 3 Instituto de Puericultura e Pediatria Martagão Gesteira (IPPMG), Universidade Federal do Rio de Janeiro, Rio de Janeiro, Brazil; 4 Department of Woman and Child Health, University of Padua, Padua, Italy; 5 Hospital General de Niños Pedro de Elizalde, Unidad de Reumatología, Buenos Aires, Argentina; 6 Hôpital Universitaire Hautepierre, Pédiatrie I, Strasbourg, France; 7 Pediatric Rheumatology Unit, Karolinska University Hospital, Stockholm, Sweden; 8 IRCCS Burlo Garofolo, Institute for Maternal and Child Health, Trieste, Italy; 9 Departamento de Pediatria, Faculdade de Ciências Médicas, Universidade Estadual de Campinas, Campinas, Brazil; 10 Clinica Pediatrica, Università degli Studi di Torino, Torino, Italy; 11 Médecine Infantile, Centre Hospitalier Universitaire Grenoble-Alpes (CHU de Grenoble), Grenoble, France; 12 Service de Pédiatrie, Hôpital de l’Archet, Nice, France; 13 Department of Pediatrics, Bērnu Klīniskā Universitātes Slimnīca, Riga, Latvia; 14 Afdelingkindergeneeskunde, Academisch Ziekenhuis Leiden, Leiden, Netherlands; 15 Meir Medical Centre, Pediatric Rheumatology Unit, Department of Pediatrics, Kfar Saba and Sackler School of Medicine, Tel Aviv University, Tel Aviv, Israel; 16 Department of Pediatric Immunology and Rheumatology, Faculty of Medicine, University of Nis, Nis, Serbia; 17 Clinic of Pediatrics, Department of Pediatric Rheumatology, Clinical Center Nis, Nis, Serbia; 18 IRCCS Istituto Giannina Gaslini, Clinica Pediatrica – Reumatologia, Genoa, Italy; 19 Dipartimento di Neuroscienze, Riabilitazione, Oftalmologia, Genetica e Scienze Materno-Infantili (DiNOGMI), Università degli Studi di Genova, Genoa, Italy

**Keywords:** Juvenile dermatomyositis, Prednisone tapering, Glucorticoids, Disease activity, Core set measures

## Abstract

**Background:**

Prednisone (PDN) in juvenile dermatomyositis (JDM), alone or in association with other immunosuppressive drugs, namely methotrexate (MTX) and cyclosporine (CSA), represents the first-line treatment option for new onset JDM patients. No clear evidence based guidelines are actually available to standardize the tapering and discontinuation of glucocorticoids (GC) in JDM. Aim of our study was to provide an evidence-based proposal for GC tapering/discontinuation in new onset juvenile dermatomyositis (JDM), and to identify predictors of clinical remission and GC discontinuation.

**Methods:**

New onset JDM children were randomized to receive either PDN alone or in combination with methotrexate (MTX) or cyclosporine (CSA). In order to derive steroid tapering indications, PRINTO/ACR/EULAR JDM core set measures (CSM) and their median absolute and relative percent changes over time were compared in 3 groups. Group 1 included those in clinical remission who discontinued PDN, with no major therapeutic changes (MTC) (reference group) and was compared with those who did not achieve clinical remission, without or with MTC (Group 2 and 3, respectively). A logistic regression model identified predictors of clinical remission with PDN discontinuation.

**Results:**

Based on the median change in the CSM of 30/139 children in Group 1, after 3 pulses of methyl-prednisolone, GC could be tapered from 2 to 1 mg/kg/day in the first two months from onset if any of the CSM decreased by 50–94%, and from 1 to 0.2 mg/kg/day in the following 4 months if any CSM further decreased by 8–68%, followed by discontinuation in the ensuing 18 months. The achievement of PRINTO JDM 50–70-90 response after 2 months of treatment (ORs range 4.5–6.9), an age at onset > 9 years (OR 4.6) and the combination therapy PDN + MTX (OR 3.6) increase the probability of achieving clinical remission (*p* < 0.05).

**Conclusions:**

This is the first evidence-based proposal for glucocorticoid tapering/discontinuation based on the change in JDM CSM of disease activity.

**Trial registration:**

Trial full title: Five-Year Single-Blind, Phase III Effectiveness Randomized Actively Controlled Clinical Trial in New Onset Juvenile Dermatomyositis: Prednisone versus Prednisone plus Cyclosporine A versus Prednisone plus Methotrexate. EUDRACT registration number: 2005–003956-37. Clinical Trial.gov is NCT00323960. Registered on 17 August 2005.

## Background

Juvenile dermatomyositis (JDM) is a rare, autoimmune disease, primarily characterized by muscle and skin involvement. Less frequently, other systems like the gastrointestinal tract or lungs may be affected. Beside the significant decrease in the mortality rate of the disease in the last years, due to the introduction of glucocorticoids (GC) and disease modifying anti-rheumatic drugs (DMARDs), disease and drug related morbidity are still a major problem [1, 2]. Therapeutic approaches for adult patients with DM are not standardized [3–5], while those for children are essentially based on consensus and literature revision [6–10]. While GC still remain the mainstay of initial and long-term treatment in new-onset JDM despite their known adverse effects, a still open question is how to taper and discontinue GC in JDM patients. A recent randomized trial in new-onset untreated JDM, conducted by the Paediatric Rheumatology International Trials Organisation (PRINTO) [11, 12], showed that combined therapy with prednisone (PDN) and either methotrexate (MTX) or cyclosporine (CSA) was more effective than PDN alone. The PRINTO trial foresaw a consensus plan for GC tapering up to discontinuation which the participating centres could follow in clinical practice for the children enrolled in the study.

The primary objective of the present study was to provide an evidence-based proposal for GC tapering/discontinuation in new onset JDM patients, through the analysis of the PRINTO JDM trial. The secondary objective of the study was to identify predictors of GC discontinuation and clinical remission (CR) on medication.

## Methods

### Patients and study design

Data on new onset JDM children from the international, multicentre, randomised, open label, superiority PRINTO trial, whose details are available elsewhere, were analysed [11, 13]. In brief, children aged 18 years or younger, with newly diagnosed (PDN higher than 1 mg/kg for no more than 1 month allowed) probable or definite JDM, as per Bohan and Peter criteria [14, 15], were included in the trial. Major exclusion criteria were the presence of cutaneous or gastrointestinal ulceration or JDM-related pulmonary disease or cardiomyopathy. Patients were considered untreated if they received one month or less of PDN, no CSA or MTX.

Patients were randomized into 3 arms to either receive PDN alone, PDN plus CSA (PDN + CSA), or PDN plus MTX (PDN + MTX). The trial was divided into three parts: induction (first 2 months), maintenance (22 months), and extension (at least 3 years). The study database was locked after the last randomized patient had completed the induction and maintenance phases.

The study was firstly approved by the ethics committees/institutional review board of the main centre in Genoa, Italy (IRCCS Istituto Giannina Gaslini, Decision nr. 77 of 09/02/2006) and then by those of all participating centres in the trial (54 centres in the following countries: Argentina, Belgium, Brazil, Czech Republic, Denmark, France, Germany, Greece, Israel, Italy, La Reunion, Latvia, Mexico, Netherlands, Norway, Serbia, Slovakia, Slovenia, Sweden, United Kingdom, USA, Venezuela).

### Glucocorticoid tapering/discontinuation PRINTO protocol

Before randomization to one of the 3 aforementioned arms, all children received three daily pulses of intravenous methylprednisolone (30 mg/kg per pulse, for a maximum amount of 1 g per pulse). In the induction phase, a consensus-based schema (NR, AR, AM and PRINTO members) suggested to administer 2 mg/kg per day of PDN or its equivalent (maximum 60 mg/day) divided in three doses per day (oral preferentially) for 1 month, then moved to morning daily dose, tapering the dose by 0.25 mg/kg every week to reach a daily dose of 1 mg/kg per day at the end of month 2. In the following 4 months PDN was supposed to be gradually tapered, as long as the patient remained clinically stable, up to a safe daily dose of 0.2 mg/kg by the end of month 6, which was maintained until the end of month 12. Afterwards, the dose of PDN was reduced to 0.1 mg/kg per day for further 6 months and then administered every other day until month 24. If a patient reached the status of inactive disease before month 24, PDN could be discontinued at physician’s discretion after discussion with the family. After the second year, treatment was at the discretion of the treating clinician.

### Assessment and outcome

Clinical assessments to define patients’ response to therapy according to PRINTO criteria were performed every two months in the initial 6 months (monthly for safety) and then every 6 months up to year 2. Treatment failure (TF) was defined as the addition of CSA or MTX or any other DMARDs in any of the 3 groups or a major increase in their dose, or major increase in PDN dose or discontinuation of the assigned therapy for any reason (adverse events [AE], lost to follow-up, etc.).

Children were defined as *responders* if they demonstrated ≥20% (or 50/70/90%) improvement in ≥3 of the 6 variables of the JDM core set [16] with ≤1 variable worsening by > 30% (muscle strength excluded) [17, 18]. The 6 validated JDM PRINTO/American College of Rheumatology (ACR)/European League Against Rheumatism (EULAR) disease activity core set variables were: [16] the Childhood Myositis Assessment Scale (CMAS) (0 = worst; 52 = best); [19] physician’s global assessment (Physician global) of the patient’s overall disease activity on a 0–10-cm visual analogue scale (VAS) (0 = best; 10 = worst); [20] the Disease Activity Score (DAS) (0 = best; 20 = worst); [21] the cross-culturally adapted and validated version of the Childhood Health Assessment Questionnaire (C-HAQ) (0 = best; 3 = worst) [22, 23]; the parent’s global assessment of the child’s overall patient’s well-being (Parent Global) on a 10-cm VAS (0 = very well; 10 = very poor) [20, 22, 23]; the parent version of the physical summary score (PhS) of the Child Health Questionnaire (CHQ) [23, 24], with lower score indicating worse quality of life.

Patients were classified as being in a status of inactive disease if they met the validated PRINTO criteria for clinically inactive disease, which means at least 3 out of the following 4 criteria: creatine kinase ≤150, CMAS≥48, Manual Muscle Testing (MMT) ≥78, and PhyGlo VAS ≤ 0.2. We then verified our results through the proposed revised criteria by Almeida et al., which require mandatory PhyGlo VAS ≤ 0.2 [25, 26]. Clinical remission was defined as a status of inactive disease maintained for 6 continuous months on medication (clinical remission on medication) or 12 continuous months off any DMARDs/glucocorticoids (clinical remission off medication) [25].

**Fig. 1:**
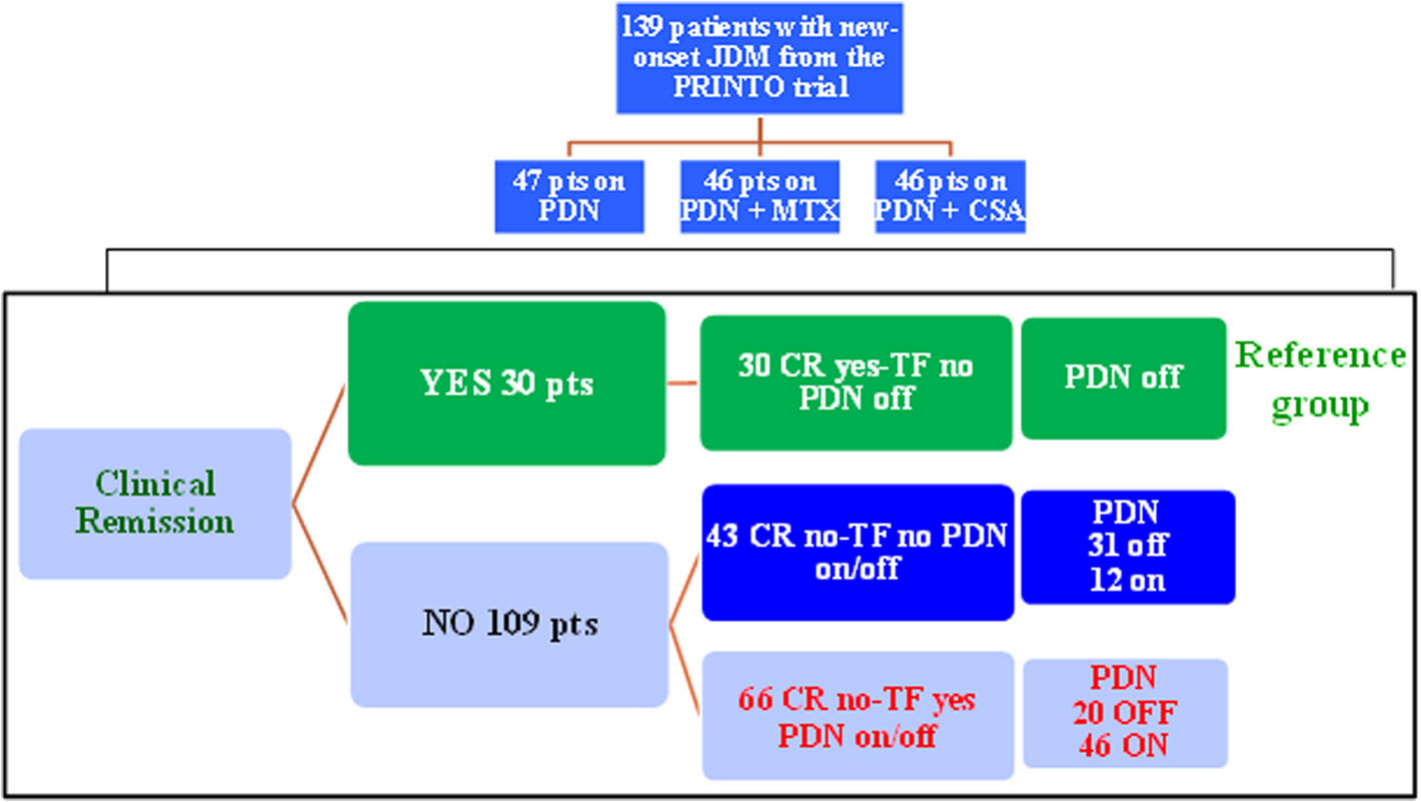
Study design. *JDM* juvenile dermatomyositis, *PDN* prednisone, *MTX* methotrexate, *CSA* cyclosporine, *pts* patients, *CR* clinical remission, *TF* treatment failure

Children in the trial were subsequently categorized into 3 mutually exclusive groups. Group 1 represented the reference standard for the best clinical outcome defined as those children on clinical remission on medication, with no TF, and no major deviation (±0.2 mg/kg PDN dose) from the assigned GC protocol, and who could discontinue PDN as per the suggested tapering schedule (CR Yes-TF no-PDN off). Group 1 was compared with those who did not achieve clinical remission, without TF (Group 2: CR no-TF no-PDN on or off) or with TF (Group 3: CR no-TF yes-PDN on or off). (Fig. 1).

### Statistical analysis

Baseline and follow-up characteristics were summarized using descriptive statistics primarily by median (1st-3rd quartiles) changes in the PRINTO core set measures (CSM) in the first 6 and over 24 months. Proportions were analyzed by chi-square, or Fisher’s Exact test while for continuous variables t-test or ANOVA. Non parametric ANOVA (Kruskal-Wallis test to compare 3 groups and Friedman test to compare repeated measures during time) has been applied in case of ordinal or non-normally distributed variables. For multiple hypothesis testing, Bonferroni’s correction was applied ([1-((1-p)^n^)], with *n* = 3 posterior comparisons).

For each core set variable, absolute change and “relative” percent change were calculated; the term “relative” refers to the fact that whenever the measure assumed the value of 0 at starting point, the percent change was calculated taking into account the range of the scale (example: the “relative” percent change of a measure changing from 0 to 2 on a 0–10 point-scale was equal to + 20% instead of the rough usual value of + 100%) [27]. When missing data occurred for spare data among the different CSM, a mean value was calculated in case the missing value was between two available time points.

The association between the change in each core set variable, and the attainment of a status of GC discontinuation or CR on medication, were analyzed by multiple logistic regression, which used all significant variables in Table 1 and the different levels of PRINTO criteria for response at months 2, 4 and 6 to calculate the baseline-to-2-month change and the baseline-to-6-month change in each core set variable and as the dependent outcome clinical remission. Odds ratios (OR) with 95% confidence intervals (95% CI) were reported. Continuous variables were dichotomized according to the best cut-offs provided by the ROC analysis. [28] The pre-analysis hypothesis was that children able to demonstrate an earlier improvement in the PRINTO CSM or PRINTO criteria of response were more likely to achieve PDN discontinuation.

**Table 1:** Baseline characteristic and PRINTO core set measures at onset of the study population

	Group 1 (CR yes-TF no-PDN off) *N* = 30	Group 2 (CR no-TF no/PDN on or off)*N* = 43	Group 3 (CR no-TF yes/PDN on or off) *N* = 66	*P* ^*^
Age at onset (years)	9.5 (6.2–12.3)	6.5 (3.3–9.8)	6.9 (4.2–10)	0.016
Disease duration (mo)	2.6 (1.3–4.7)	2.6 (1.3–6.4)	3.0 (1.5–4.8)	0.94
MD-global (0–10 ↑)	7.0 (6.0–8.0)	6.0 (5.0–7.0)	7.0 (5.0–8.0)	0.17
Parent global (0–10 ↑)	6.0 (5.0–8.0)	5.0 (3.5–7.0)	5.4 (5.0–7.0)	0.40
CHAQ (0–3 ↑)	1.8 (1.1–2.6)	1.8 (1.4–2.6)	1.9 (1.3–2.5)	0.94
DAS (0–20 ↑)	13.0 (11.0–15.0)	13.0 (11.0–15.0)	13.0 (11.0–15.0)	0.80
CMAS (0–52 ↓)	16.5 (13.0–33.0)	21.0 (14.0–35.0)	20.7 (11.0–32.0)	0.96
MMT (0–80 ↓)	40.0 (30.0–60.0)	47.0 (35.0–58.0)	48.0 (34.0–56.0)	0.84
CHQ PhS (40–60 ↓)	19.8 (9.4–33.4)	12.7 (5.2–23.5)	14.9 (8.1–22.4)	0.16

Data are medians (1st 3rdquartiles). *P: *P* value refers to the non-parametric Analysis of Variance (Kruskal-Wallis test); MD-global: physician’s global assessment of the patient’s overall disease activity on a 0–10-cm visual analogue scale (VAS); Parent global: parents’ global assessment of the child’s overall patient’s well-being on a 10-cm VAS; *DAS* Disease Activity Score; *CMAS* Childhood Myositis Assessment Scale, *MMT* manual muscle testing; *CHAQ* cross-culturally adapted and validated version of the Childhood Health Assessment Questionnaire); ↑ indicates that higher values correspond to a worse outcome; ↓ indicates that lower values correspond to a worse outcome

Data were entered into an Access XP database and analyzed with Excel XP (Microsoft), XLSTAT 6.1.9 Addinsoft, Statistica 6.0 (StatSoft, Inc), and Stata 7.0 (Stata Corporation).

## Results

As shown in Figs. 1, 139 children from 54 centres in 22 countries were enrolled and randomized in the trial. Thirty (21.6%) patients were classified in Group 1 (CR yes-TF no-PDN off), 43 (30.9%) in Group 2 (CR no-TF no/PDN on or off), and 66 (47.5%) in Group 3 (CR no-TF yes/PDN on or off). At baseline all the three groups had a high level of disease activity with no differences in the CSM (Table 1). Age at onset was the only variable with a statistically significant difference at baseline among the three groups with children in Group 1 (CR yes-TF no-PDN off) showing an older age at onset (9.5 versus 6.5 versus 6.9, *p* = 0.016).

Most of the patients in Group 1 (15 pt., 50%) received MTX + PDN, most of the patients in group 3 (30 pt., 45.5%) received PDN alone, while Group 2 was well balanced between the different drugs (9 pt. – 20.9% received PDN, 14 pt. – 32.6% PDN + MTX and 20 pt. – 46.5% PDN + CSA) in the middle (data not shown).

### Change over time in PRINTO core set measures

**Fig. 2:**
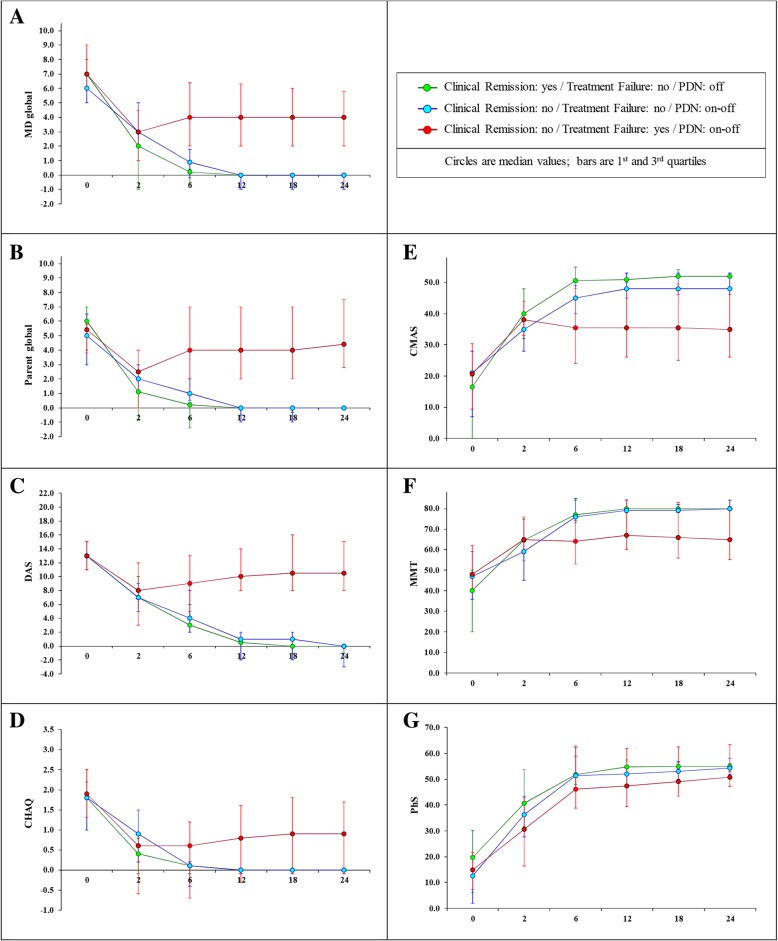
Trend of the PRINTO CSM and MMT in the 3 groups of children with JDM. [A = MD-global: physician’s global assessment of the patient’s overall disease activity on a 0–10-cm visual analogue scale (VAS); B=Parent global: parents’ global assessment of the child’s overall patient’s well-being on a 10-cm VAS; C = DAS: Disease Activity Score (range 0–20); D = CHAQ: cross-culturally adapted and validated version of the Childhood Health Assessment Questionnaire (range 0–3); E = CMAS: Childhood Myositis Assessment Scale (range 0–52); F = MMT: manual muscle testing (range 0–80); G = PhS: physical summary score of the Child Health Questionnaire (range 40–60)]

Figure 2 shows the trend over time of the PRINTO CSM and MMT in the 3 groups. Already after 2 months of treatment the 3 groups start to differentiate for all the disease activity measures in comparison to baseline. A statistically significant trend over time can be recognized from month 4 especially between Group 1 (CR yes-TF no-PDN off) and Group 3 (CR no-TF yes-PDN on or off), in particular for MD global, parent global, DAS, CMAS, MMT (all *p* values < 0.001) and CHAQ (p value = 0.002), with the exception of PhS that is very close to statistical significance and starts to be significant from month 12 onward.

When we considered patients in inactive disease according to the two different definitions (PRINTO criteria [25] and Almeida’s criteria [26]), a complete overlap could be identified, since all the 30 patients from Group 1 (CR yes-TF no-PDN off) met both definitions having a Physician VAS ≤ 0.2.

Analyzing in more details the first 6 months from treatment start, while Groups 1 and 2 showed a similar trend in the improvement of disease activity parameters in the initial 2 to 6 months, Group 2 was not able to reach a status of clinical remission despite PDN being discontinued in 31/43 (72%) of the patients. Group 3 was even more different since only 20/66 (30%) were able to discontinue GC despite a TF which required a major change in treatment to control disease activity parameters (Figs. 1 and 2).

Table 2 shows the analysis of the median absolute change and relative percent change in disease activity parameters for the patients in the reference Group 1 (CR yes-TF no-PDN off). The median change in the CSM of Group 1 (CR yes-TF no-PDN off) was more pronounced in the initial 6 months of treatment (Table 2). In particular, an at least 50% change was present in all CSM already at month 2, with a marked CMAS change rising to 94% at month 2, and MD-global, MMT or DAS change of at least 3, 20 and 7 units, respectively. After 2 months from randomization and treatment start, the change in CSM became much less pronounced, with stable values after 6 months. With respect to the range of the scale for each core set variable, there was a considerable relative percent change in the different CSM over time (Table 2).

**Table 2:** 24 month-change in the PRINTO Core Set Measures in Reference Group 1 (N = 30)

	0 months	0–2 months Absolute change (% change)	2–4 months Absolute change (% change)	4–6 months Absolute change (% change)	6–24 months Absolute change (% change)
MD evaluation (0–10 ↑)	7	-3 (− 66.7%)	−1 (− 68.3%)	0 (0%)	0 (0%)
DAS (0–20 ↑)	13	−7 (− 50%)	−2 (− 40%)	−1 (− 33.3%)	−1 (− 6.5%)
CMAS (0–52 ↓)	16.5	+ 16 (+ 93.8%)	+ 4 (+ 15.8%)	+ 1 (+ 2.0%)	+ 1 (+ 3.4%)
MMT (0–80 ↓)	40	+ 20 (+ 53.8%)	+ 6 (+ 8.1%)	+ 1.5(+ 2.1%)	+ 1 (+ 1.3%)
Parent global (0–10 ↑)	6	−4 (− 76.4%)	0 (0%)	0 (0%)	0 (0%)
CHAQ (0–3 ↑)	1.8	−1.2 (−82.5%)	−0.1 (− 28.6%)	0 (0%)	0 (0%)
PhS (40–60 ↓)	19.8	+ 14.2 (+ 53.8%)	+ 8.4 (+ 21.1%)	+3.2 (+ 7.3%)	+ 1.9 (+ 4.3)

Group 1: clinical remission yes- treatment failure no- prednisone off. MD-global: physician’s global assessment of the patient’s overall disease activity on a 0–10-cm visual analogue scale (VAS); Parent global: parents’ global assessment of the child’s overall patient’s well-being on a 10-cm VAS; *DAS* Disease Activity Score, *CMAS* Childhood Myositis Assessment Scale, *MMT* manual muscle testing, *CHAQ* cross-culturally adapted and validated version of the Childhood Health Assessment Questionnaire; ↑ indicates that higher values correspond toa worse outcome; ↓indicates that lower values correspond to a worse outcome

### Predictors of glucocorticoid discontinuation in children with clinical remission on medication

The logistic regression model, including age at onset (the only statistically significant variable in Table 1), and the different levels of PRINTO criteria for response at months 2, 4 and 6, showed that the achievement of a PRINTO JDM 50–70-90 response at 2 months (OR range 4.5–6.9) from treatment start, an age at onset > 9 years (OR 4.6) and the combination therapy PDN + MTX (OR 3.6) increase the probability of achieving CR (*p* < 0.05) (Table 3).

**Table 3:** Predictors of clinical remission and prednisone discontinuation

	Odds Ratio (95% CI)	*p* ^#^
Responder at 2 months:		
Printo-50 (vs. not responder/Printo-20)	5.41 (1.37–21.32)	0.0076
Printo-70 (vs. not responder/Printo-20)	6.90 (1.91–24.99)	
Printo-90 (vs. not responder/Printo-20)	4.46 (1.08–18.38)	
Onset age > 8.53 years (vs. ≤ 8.53 years)	4.64 (1.69–12.71)	0.0017
Therapygroup: PDN + MTX (vs. PDN/PDN + CSA)	3.63 (1.30–10.09)	0.0116
AUC of the model	0.80	

*PDN* prednisone, *MTX* methotrexate, *CSA* Cyclosporine. *OR* Odds Ratio, *95% CI* 95% Confidence Interval; *p*^#^: Likelihood Ratio test

### The PRINTO evidence-based glucocorticoid tapering/discontinuation proposal

**Fig. 3:**
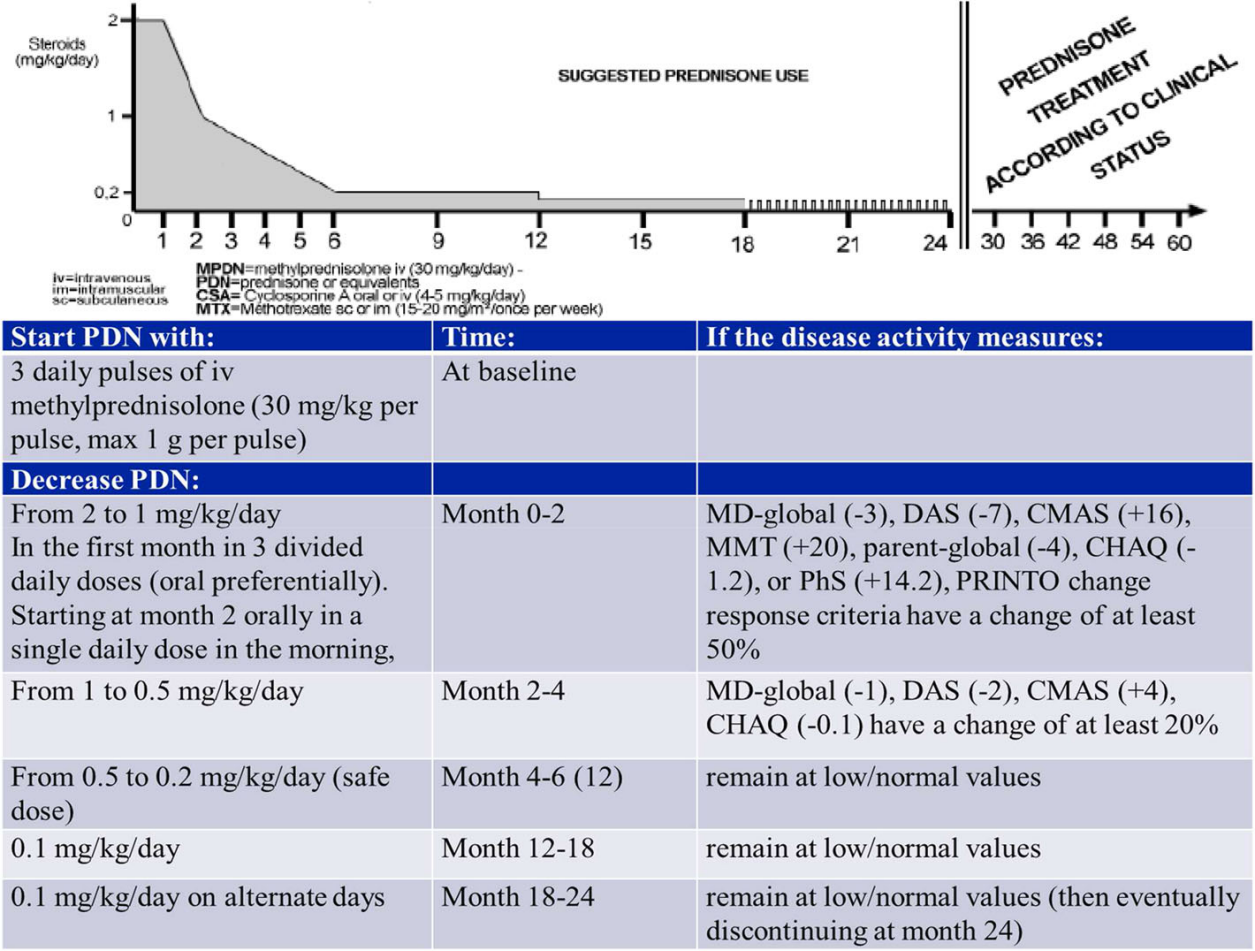
PRINTO evidence-based proposal for PDN tapering/discontinuation in a 2-year time frame

Figure 3 shows the PRINTO evidence-based treatment plan for GC tapering/discontinuation in children with new onset JDM. In brief, after 3 pulses of methyl-prednisolone, PDN could be tapered from 2 to 1 mg/kg/day in the first two months from onset if any of the CSM decrease by at least 50%, and from 1 to 0.2 mg/kg/day in the following 4 months if any of the CSM decreases further by 8–68%, followed by discontinuation over the next 18 months.

All children in group l followed the PDN consensus protocol for tapering treatment, as opposed to 5/43 (11.6%) and 50/60 (75.8%) in groups 2 and 3, respectively.

## Discussion

The PRINTO JDM trial was a randomized study with a considerable number of new-onset JDM patients suited to provide an evidence-based proposal for GC tapering/discontinuation. It demonstrated that the use of combined therapy, MTX + PDN, is the best treatment option in these patients in terms of safety and efficacy. The PRINTO consensus-based steroid tapering protocol proposed in the randomized trial was tested against the strongest outcome of clinical remission off GC to extrapolate an evidence-based proposal for GC tapering/discontinuation.

At baseline, disease activity was comparable in all the three groups of patients analyzed, but the group of patients with an older age showed a better outcome as confirmed by the logistic regression model. After treatment initiation, the three groups significantly differentiated over time, with a clear trend given by a sharp decrease in disease activity parameters, which was particularly evident when the reference Group 1 was compared with the other two groups. Most important, the differentiation in disease activity started very early (already after 2 months), and became apparent within the first 6 months of observation. This means that a trend toward a fast decrease in disease activity parameters can be recognizable in the early phase of treatment and can be used as clinical predictor of a better outcome. While group 1 and 2 showed a similar trend in the decrease of disease activity parameters in the initial 2 to 6 months, they represent a different clinical phenotype according to clinical remission, so they could not be combined. Group 3 had the worst outcome since just one third was able to discontinue glucocorticoids, but with the need to introduce a major change in treatment to control disease activity.

The analysis was therefore focused on Group 1 (CR yes, TF no, PDN off), as the reference standard group in the trial population. Indeed this group showed a greater improvement in disease activity, which was already evident after 2 months. Based on the observation of this group, we could provide an evidence-based proposal on how to taper prednisone, up to discontinuation, in new onset JDM patients using quantitative cut-offs of CSM, that foresee a rapid decrease in few months to the dose of PDN which is thought to limit effects on growth [29]. Indeed, the knowledge of lower cut-offs for the single core set parameters, whose achievement may allow the clinician to modify therapy, is a practical indicator for the physician who has to decide in the everyday clinical practice how to use the JDM outcome measures to taper GC.

In the last years, literature provided consensus-based recommendations, aimed to facilitate diagnosis and treatment of JDM patients through multiple meetings among clinicians and researchers. In this context the Children’s Arthritis and Rheumatology Research Alliance (CARRA) proposed consensus treatment plans for the management of JDM patients with recent onset [9, 30]. The use of GC in JDM was a main point of the consensus, with the specialists providing indications on the timing and modality of steroid tapering and use. Despite the longest period for GC discontinuation proposed by this study, and differently from the CARRA recommendation, we think that it can be a valuable option for GC tapering/discontinuation due to the evidence of strong outcome (clinical remission off GC) in the PRINTO cohort, the already reported acceptable safety profile [11, 12], and the possibility to achieve after only 6 months the safe dose of 0.2 mg/kg/day, so avoiding long PDN exposure and subsequent side effects. Moreover, the PRINTO JDM trial represents a large source of prospective data on a very rare condition in pediatrics, which still needs to be clarified in multiple aspects. This allows observations on real patients’ data with the advantage of reporting evidence-based information. Differently from CARRA recommendations, nevertheless, our treatment plan provides evidence that it is possible to achieve the steroid dose of 0.2 mg/kg/day, considered safer especially for growth impairment, within 6 months instead of 9, with good clinical control, so avoiding long PDN exposure and subsequent side effects. In 2016, a similar effort, conducted by a European initiative called Single Hub and Access point for pediatric Rheumatology in Europe (SHARE) initiated in 2012 to provide diagnostic and therapeutic regimens for all the pediatric rheumatic diseases, yielded, through a consensus process, recommendations on diagnosis and therapy in JDM, including consensus on steroid use, to be reduced as the patient shows clinical improvement [10]. The PRINTO proposal for tapering will allow pediatric rheumatologist to take advantage of concrete quantitative indications about how to use GC and when and how to taper them in clinical practice based on the change in individual CSM. The importance of evaluating quantitatively CSM in the JDM patient follow-up is consequently a major point and should be performed by all the pediatric rheumatologists at each visit [16–18, 31–40].

The results of this study are important also in light of future treatment plans which apply a treat-to-target strategy, already proposed in the management of juvenile idiopathic arthritis (JIA) [41]. Indeed, the identification that the attainment of at least JDM PRINTO 50 criteria of response in 2 months or an improvement by at least 50% in CSM (90% in CMAS), especially in children treated with the combination of PDN + MTX, is a very early predictor of clinical remission and steroid discontinuation, favors the hypothesis that earlier and more aggressive treatment might lead to a better outcome. The results of this study might allow the pediatric rheumatologists to tell patients and families if there is a higher probability of achieving remission according to the course of the disease in the first months from treatment start. Looking from a different perspective a patient without these characteristics could be considered at higher risk of a worse disease course and deserve therefore more aggressive treatments.

This work has some limitations that should be considered by the pediatric rheumatologist, willing to follow our proposal. First of all, a limitation of our study may be represented by the long period of administration of steroids, whose side effects may prevent the use of this medication. Nevertheless, the cumulative dose of PDN per kilo of body weight was 250.88 mg for both group 1 and 2, and 250.9 for group 3, (data not shown) which means that the cumulative dose of GC was similar for all the three groups, not modifying the PDN-related side effects. Moreover, this steroid schedule allows to reach what is considered in all trials in poly-articular course JIA a safer dose of PDN (0.2 mg/kg/day or 10 mg/day whichever is lower) [42–46], in a short time (6 months), continuing just with a very low dose up to month 24, when discontinuation may be attempted. Finally, in an independent PRINTO series of 275 patients collected worldwide, 41/98 (41.8%) of children with recent onset JDM were still on glucocorticoid treatment after 24 months from disease onset. [5]

Up to now, we cannot compare our results with other randomized trials on steroid tapering/discontinuation in new- onset JDM patients. The lack of alternative steroid protocols, provided by evidence-based trials, or standard consensus on glucocorticoid therapy for both intravenous and oral induction, does not allow further considerations about safety and efficacy of the proposed steroid tapering schedule neither comparison with a shorter steroid course, likely to be considered preferable. Since the purpose of the present work was not to provide recommendations on the use of glucocorticoids in JDM, but to propose a possible protocol for glucocorticoids tapering withdrawal in new-onset JDM patients, the proposed protocol may help the pediatric rheumatologist to deal at onset with such a challenging disease.

Another major point of discussion is the presence of around 50% of patients in Group 1 on PDN + MTX, which is the most effective treatment group in the PRINTO JDM trial [11]. This could have induced an improvement in disease activity, not due to the proposed glucocorticoid tapering protocol, but to the combination therapy with MTX as also underlined by the logistic regression model. It should be noted, however, that one third of patients in the other two groups, received the same combination of PDN + MTX.

Another limit of the present work is the small comparative sample size and reference group. However this should be read in light of the rarity of the disease and the lack of prospective data on new onset JDM patients which makes our results noteworthy.

Finally, it is notable that a large percentage of patients (42%) failed to discontinue GC according to the proposed weaning schedule. If we consider that all the three groups of patients presented at baseline with the same demographic and clinical features (Table 1), except for a slightly increased disease duration in Group 3, and since MTX therapy is well-proven in efficacy in JDM patients [11], this should be read as a warning in considering MTX at the very beginning of the JDM patient history.

The lack of change in the CSM after 12 months in Group 1 and 2 in particular may raise the need of new trials testing two different strategies for steroid tapering with the main aim to shorten the on-therapy period. In fact, in order to either confirm our results or propose new treating strategies in new onset JDM patients, there is the new of new comparative studies in pediatrics.

## Conclusions

This study provides a steroid tapering plan in new-onset JDM patients. In particular, we propose evidence-based specific quantitative cut-offs for glucocorticoids tapering/discontinuation based on the change in the CSM in the initial 6 months of treatment as well as in the overall response to treatment and we identify early predictors of remission, to be used in daily practice and in future clinical trials by pediatric rheumatologists.

## Abbreviations

ACR: American College of Rheumatology
AE: Adverse event
CARRA: Children’s Arthritis and Rheumatology Research Alliance
CHAQ: Childhood Health Assessment Questionnaire
CHQ-PhS: parent version of the physical summary score
CMAS: Childhood Myositis Assessment Scale
CR: Clinical remission
CSA: Cyclosporine
CSM: Core set measure
DAS: Disease Activity Score
DMARDs: Disease-modifying anti-rheumatic drugs
EULAR: European League Against Rheumatism
GC: Glucocorticoids
JDM: Juvenile dermatomyositis
MD Global: Physician Global Activity
MMT: Manual muscle testing
MTX: Methotrexate
PDN: Prednisone
PRINTO: Paediatric Rheumatology INternational Trials Organisation
SHARE: Single Hub and Access point for pediatric Rheumatology in Europe
TF: Treatment failure
VAS: Visual analog scale
